# Amyotrophic Lateral Sclerosis After Exposure to Manganese from Traditional Medicine Procedures in Kenya

**DOI:** 10.1007/s12011-020-02501-4

**Published:** 2020-11-23

**Authors:** Elin Roos, Sebastian K.T.S. Wärmländer, Jeremy Meyer, Sabrina B. Sholts, Jüri Jarvet, Astrid Gräslund, Per M Roos

**Affiliations:** 1 Department of Global Public Health, Karolinska Institutet, 171 77 Stockholm, Sweden; 2 Department of Biochemistry and Biophysics, Stockholm University, 106 91 Stockholm, Sweden; 3 UCLA/Getty Conservation Programme, Cotsen Institute of Archaeology, UCLA, Los Angeles, CA 90095 USA; 4 Unit for Surgical Research, Medical School of Geneva, University of Geneva, 120511, 14 Genève, Switzerland; 5 Department of Anthropology, National Museum of Natural History, Smithsonian Institution, 370 12, Washington D.C, USA; 6 The National Institute of Chemical Physics and Biophysics, 12618 Tallinn, Estonia; 7 Institute of Environmental Medicine, Karolinska Institutet, 171 77 Stockholm, Sweden; 8 Department of Clinical Physiology, St. Goran Hospital, 112 81 Stockholm, Sweden

**Keywords:** Amyotrophic lateral sclerosis, Manganese, Neurodegeneration, Potassium permanganate, Traditional medicine

## Abstract

Amyotrophic lateral sclerosis (ALS) is a fatal neurodegenerative disease characterized by motor neuron loss and widespread muscular atrophy. Despite intensive investigations on genetic and environmental factors, the cause of ALS remains unknown. Recent data suggest a role for metal exposures in ALS causation. In this study we present a patient who developed ALS after a traditional medical procedure in Kenya. The procedure involved insertion of a black metal powder into several subcutaneous cuts in the lower back. Four months later, general muscle weakness developed. Clinical and electrophysiological examinations detected widespread denervation consistent with ALS. The patient died from respiratory failure less than a year after the procedure. Scanning electron microscopy and X-ray diffraction analyses identified the black powder as potassium permanganate (KMnO4). A causative relationship between the systemic exposure to KMnO4 and ALS development can be suspected, especially as manganese is a well-known neurotoxicant previously found to be elevated in cerebrospinal fluid from ALS patients. Manganese neurotoxicity and exposure routes conveying this toxicity deserve further attention.

## Introduction

Amyotrophic lateral sclerosis (ALS) is a fatal neurodegenerative disorder characterized by loss of upper and lower motor neurons and sclerosis of motor pathways in the spinal cord, leading to widespread progressive skeletal muscle atrophy [1] and death by respiratory failure. Electrophysiological examinations are necessary in the diagnostic workup of ALS, as ALS-mimicking disorders exist, most commonly in the form of myopathic conditions [2]. The ALS incidence is about 3 per 100 000 person-years [3], with a peak age of diagnosis at 54 years and a projected incidence increase in coming years [4]*.* Around 50% of the patients die within 30 months after symptom onset, while around 20% may survive up to 10 years [5]*.* Several hypotheses for ALS pathogenesis have been proposed.

The notion that increased cellular oxidative stress contributes to ALS is supported both by observations from post-mortem ALS tissues, where widespread accumulation of oxidative damage to proteins, lipids, and DNA have been noted [6], and by studies showing that superoxide dismutase (SOD1) mutations are related to increased protein and lipid oxidation [7]. A related putative ALS cause is aggregation of misfolded SOD proteins [8]. Aberrations in chromosome 9 (C9orf72) with nucleotide repeats and hyperphosphorylated tau protein observed in sporadic ALS have also been proposed as potentially causative [9], as has mRNA dysmetabolism [7]. Markers of inflammation are elevated in ALS [10], and the hypothesis that inflammation plays a causative role in ALS is strengthened by observations of activated macrophages and the presence of dendritic cells in ALS spinal cord tissue [11]. A fifth possibility is that metals with neurotoxic or/and redox-cycling properties act as triggers for oxidative stress and neuroinflammation [12, 13].

The interplay between genetic and environmental risk factors for ALS is likely complex and has been extensively discussed for several decades. Cigarette smoking [14], pesticide exposure, and advanced age are known risk factors [15, 16], and a recent thorough meta-analysis of ALS occupational risk factors brought metal exposure to the fore [17]. Manganese (Mn) [18] and lead (Pb) [19] have been specifically implicated in ALS pathogenesis [20, 21]. Adding to this search for causative factors in ALS, we present here a case of a patient who developed ALS shortly after subcutaneous exposure to a metal powder during a traditional medicine procedure in Kenya.

## Case Report

**Fig. 1:**
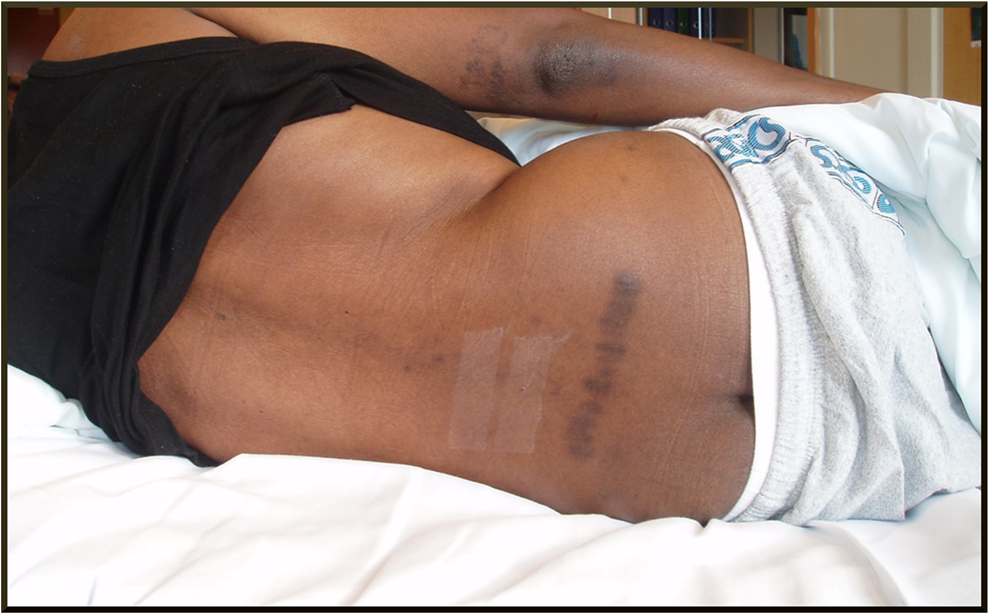
The studied patient with multiple parallel lumbar scars from the ogosaraka scarification procedure. Photo by P.M.R

**Fig. 2:**
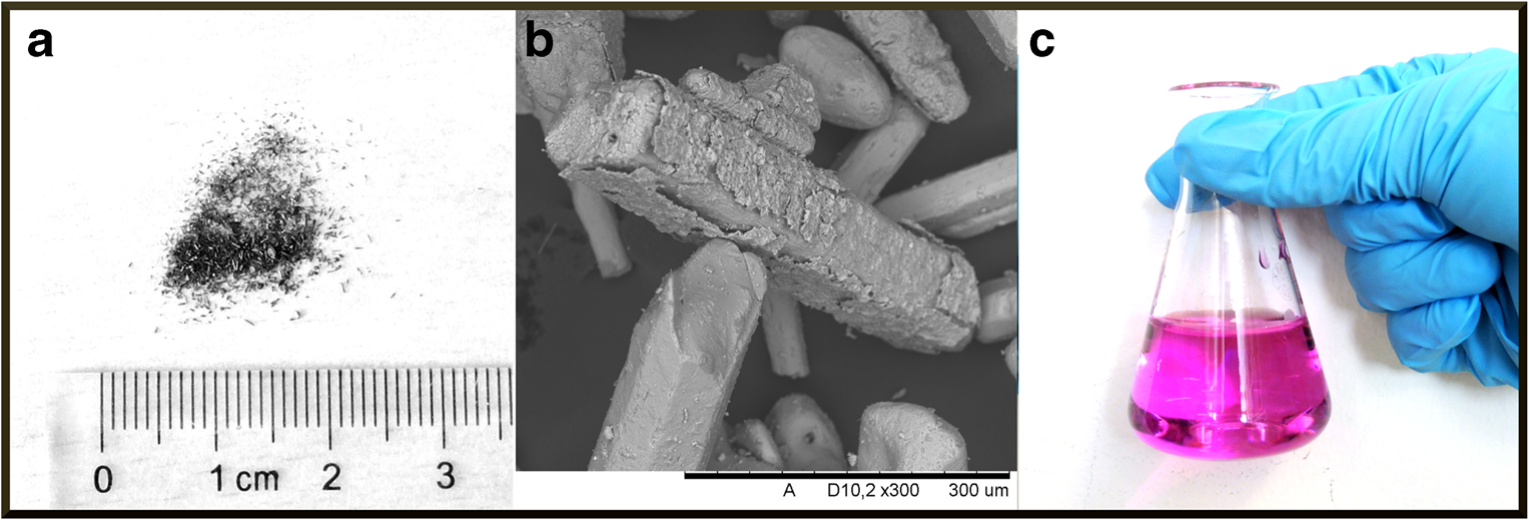
a Photograph of the black powder particles. b SEM image of the black powder particles at 300 x magnification. c Photograph of the black powder dissolved in water. Images by S.K.T.S.W

A 56-year-old previously healthy military photographer from the Kisii region in Kenya sought medical attendance for lower back pain. After 8 months of unsuccessful treatments with non-steroidal anti-inflammatory drugs, he consulted a local spiritualist and herbalist in Kisii. This region is known for its long history of active use of traditional medicine, defined as “the knowledge, skills and practices based on the theories, beliefs and experiences indigenous to different cultures, used in the maintenance of health and in the prevention, diagnosis, improvement or treatment of physical and mental illness” [22]. The patient then underwent a traditional medicinal procedure involving lower back scarification known as *Ogosaraka* in the local Kisii language (personal communication from the patient’s family). Along a line connecting the posterior iliac spines in the lumbar region, several sagittal cutaneous incisions were cut in parallel with a sharp razor blade (Fig. 1), and a glistening black metal powder (Fig. 2a) was inserted into the open wounds.

About 4 months after the scarification procedure, the patient still suffered continuous lumbar pain, and magnetic resonance imaging found a herniated lumbar disc at level L4/L5 and L5/S1. Symmetrical muscle weakness in the legs, arms, and neck developed slowly followed by head drop. Progressively he developed widespread muscle weakness and could hardly walk or carry heavy objects. He had difficulties coughing, lost weight, and displayed distinct generalized muscle atrophy. Routine bloodwork was normal, and a liver ultrasonography performed due to slightly elevated liver enzymes was also reported as normal. About 7 months after the scarification procedure, the patient sought healthcare at Oslo University Hospital in Norway, where a complete neurological assessment including a clinical neurological examination performed by a specialist in neurology indicated ALS. A neurophysiological examination with neurography and electromyography showed fibrillation potentials and positive sharp waves in several investigated skeletal muscles, including the glossus muscle, and concluded with widespread axonal damage and denervation consistent with ALS. The ALS diagnosis was confirmed by two specialists in neurology. The patient died from respiratory failure about 10 months after the scarification procedure.

## Materials and Methods

A sample of a metal powder used in the scarification procedure was collected by the patient’s daughter and transferred from Kenya to the Department of Biochemistry and Biophysics at Stockholm University in Sweden for analysis. A Hitachi TM-3000 scanning electron microscope (SEM), operating at 15 kV and equipped for elemental analysis via energy-dispersive spectroscopy (SEM–EDS), was used to characterize the shape and chemical composition of the powder particles. These investigations were performed at high vacuum (10^-5^ Torr) without surface coating or other sample preparation. An X’Pert-PRO X-ray powder diffractometer from PANalytical B.V. (The Netherlands), operating at 40 mA/45 kV and running the sample for two hours, was used to record X-ray diffraction (XRD) data for the powder. The SEM data was analyzed using the Quantax 70 software (Bruker, Germany), while the X-ray diffractogram was processed and analyzed using the X’Pert Data Viewer software (PANalytical, The Netherlands).

## Results

**Fig. 3:**
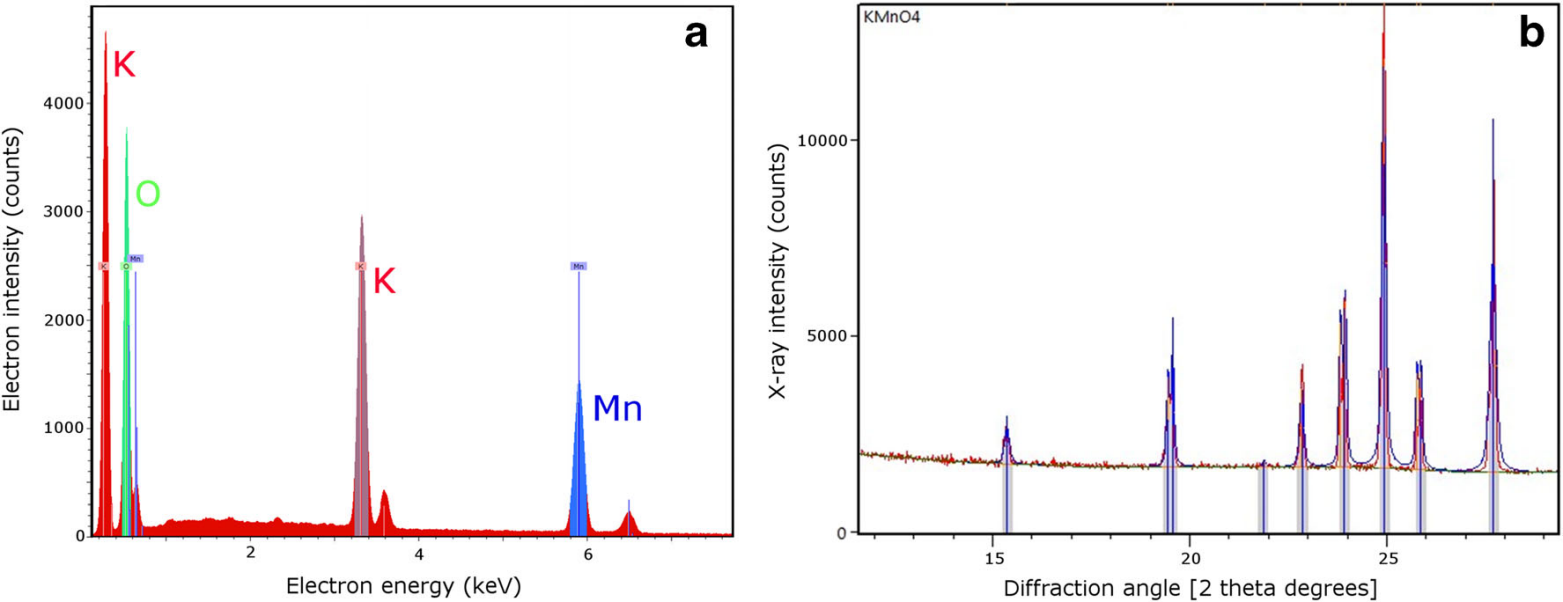
a SEM-EDS spectrum of the black powder, identifying the elements K, Mn, and O. **b** X-ray diffractogram of the black powder. The peaks in the spectrum correspond to the angles where constructive diffraction interference occurs. The observed peaks perfectly match the reference diffraction angles for KMnO4 crystals (blue lines). Images by S.K.T.S.W

Scanning electron microscopy images showed that the black glistening powder (Fig. 2a) inserted into the lower back incisions of the patient consisted of particles in the shape of small rods (Fig. 2b). Scanning electron microscopy-energy-dispersive spectroscopy (SEM-EDS) spectra identified the elements K, Mn, and O in these rods, roughly in the proportion 1:1:4 (Fig. 3a). When dissolved in distilled water, the black powder produced a solution of violet color (Fig. 2c). These observations taken together indicated that the powder was pure potassium permanganate (KMnO_4_). This was confirmed by XRD analysis, where the recorded diffractogram showed a perfect match with reference data for KMnO_4_ crystals (Fig. 3b). The latter analysis confirmed also that even though SEM-EDS analysis is useful when characterizing unknown materials [23–28], it is only the combination with XRD analysis that allows for unambiguous identifications of inorganic compounds [29–33].

## Discussion

Metal exposure seems to potentially contribute to ALS pathogenesis [20]. Redox-active ions of metals such as Cu [34], Fe [35], Mn [18], and Pb [15] appear to be particularly harmful. Such metal ions may act as triggers for cellular oxidative stress, hyperphosphorylation of the tau protein [36], and/or inflammation. To what extent these complex disease mechanisms are induced by metal exposure in the case of ALS remains to be clarified.

The patient was subjected to a scarification procedure where a black powder consisting of pure potassium permanganate (KMnO_4_; Figs. 2 and 3) was inserted into the fresh cuts. In sub-Saharan Africa, it is not uncommon to insert foreign substances into dermal cuts to achieve scarification patterns similar to tattoos [37, 38]. Scarification can however also be a medical procedure, either as a treatment in itself, or as a way to introduce various substances into the patient’s circulation by rubbing them into the wound [23, 39, 40]. Some of these substances, such as toxic minerals or medicinal plants containing heavy metals, may have negative health effects [23, 41]. Identifying health risk factors in traditional practices is a growing field of research [23, 42, 43] of particular importance in Africa, where most people use both modern (Western) and traditional African healthcare [44], where the traditional substances used are largely undocumented [23] and where advocates of the right to choose traditional medicine are gaining momentum [45].

KMnO_4_ is a well-known disinfectant and a core item on the World Health Organization (WHO) Model List of Essential Medicines [46]. As an antiseptic agent in dermatological medicine, KMnO_4_ as a dilute aqueous solution is recommended in the healing of suppurating wounds [47]. It does not occur naturally as a salt or mineral but was conceived and patented as a novel chemical disinfectant in the 1850s [48]. Although not a traditional compound, KMnO_4_ in crystalline form is today widely used by African traditional health practitioners (THPs) [23]. In a recent South African study [49], 99 THPs reported using KMnO_4_ for patients with skin rash or wounds, 74 used it for aches, 23 for sexually transmitted diseases, and 23 for nervous conditions. The main administration routes were in baths (*n* = 94), by oral ingestion (n = 67), and in herbal compresses (*n* = 66), although 25 THPs described that they used KMnO_4_ through subcutaneous implantation. Sociocultural uses of KMnO_4_ are generally unregulated and not commonly reported [49], but several hospitals in South Africa have documented KMnO_4_ misuse, often with detrimental effects [50–53].

Chemically, KMnO_4_ is a very strong oxidant. In a neutral solution, the MnO_4_^-^ anion will be reduced to MnO_2_, while acidic or reducing environments allow full reduction to a free Mn^2+^ ion, corresponding to an uptake of five electrons [54]. The large oxidizing capacity of the MnO_4_^-^ anion is the main rationale for its toxicity [55–57]. In addition to the direct oxidative damage induced by MnO_4_^-^ anions, the end products after reduction—MnO_2_ and free Mn^2+^ ions—may also be harmful, especially as Mn^2+^ ions are capable of generating harmful oxygen radicals via Fenton-type chemical reactions [58, 59]. The Mn^2+^ ions are essential to proper nerve cell function at physiological concentrations [60] but toxic at higher concentrations [61], resulting in a U-shaped dose-response curve for Mn^2+^ toxicity [60, 62].

The profound neurotoxicity of Mn is well established [63], as it is for Pb and Cd, and Mn is considered a risk factor for ALS [15, 20]. Elevated Mn concentrations have been found in cerebrospinal fluid from patients with ALS [12, 18], and in spinal cord transverse sections from deceased ALS patients [64]. The accumulation of Mn in the spinal cord seems to be most pronounced in the anterior horn cells [65] and lateral columns [64], which are the first to degenerate in ALS. A few cases of Parkinson-like syndromes associated with the use of intravenous KMnO_4_ have been described [66].

Manganese exposure from food sources has been described to contribute to ALS [7, 67, 68], yet measurements of Mn exposures in relation to ALS are scarce. Manganese passes barriers between blood and the nervous system [69] and Mn affects mitochondria of ALS nerve cells [70, 71] specifically mitochondrial respiratory chain protein function and ATP production [72].

Oxidative damage to nerve tissues has been described in sporadic as well as in familiar ALS and also in other neurodegenerative diseases [7, 73–78]. In the present case, chronic oxidative damage originating from accumulated Mn^2+^ ions, as well as oxidative damage caused by systemic exposure to MnO_4_^-^ anions, is presumed to represent a contributing pathogenic mechanism for the ALS observed. Exposure routes leading to metal-induced toxicity of the nervous system are varied and complex [20, 60]. With the KMnO_4_ salt residing inside the well-vascularized subcutaneous tissue for several months, and possibly also in paraspinal muscles, accumulations of Mn in remote tissues may be expected. Body fluid or tissue manganese concentrations were however not measured for this patient. Retrograde axonal transport of Mn and other metals has been described in detail in both rodents [79] and humans [80]. In this case, transport of Mn from subcutaneous tissues and muscle to anterior horn cells of the spinal cord may have occurred across the blood-spinal-cord-barrier (via the bloodstream) and/or via retrograde axonal transport [80].

The average survival time from diagnosis of ALS is about 3 years [15]. In this case, a rapid disease progression was noted after exposure to KMnO_4._ To what extent the Mn exposure contributed to this rapid disease progression is unknown. We have described possible exposure routes and toxicity mechanisms for Mn. If future studies support the possibility of a causal link between Mn exposure and ALS, it might become relevant to discuss treatments against metal exposure such as chelation therapy [81–83]. Other metals with potential relevance for ALS pathogenesis are Cu, Al, As, Cd, Co, Zn, V, and U, all of which have been found in significantly elevated concentrations in the CSF of ALS patients, compared to healthy controls [20, 21]. Taken together, the presented findings suggest that the use of minerals and metal salts such as KMnO_4_ in African traditional medicine can be directly harmful to the patient are consistent with a connection between ALS, Mn exposure and oxidative damage, and highlight the risks involved in using undocumented chemicals in traditional medical practices.

## Conclusions

Manganese exposure is a possible risk factor for ALS. Further studies on neurotoxic metals in ALS pathogenesis are warranted.
